# Predicting the Success of Ultrasound-Guided Supraclavicular Brachial Plexus Block Using Pulse Oximeter Perfusion Index

**DOI:** 10.7759/cureus.106978

**Published:** 2026-04-13

**Authors:** Hashim R Ameeruddin, Narayanan Unnithan, Asma H Thanduparackal, Sree Rekha AR

**Affiliations:** 1 Anesthesia and Critical Care, Kasturba Medical College, Manipal, Manipal, IND; 2 Anesthesia and Critical Care, Yenepoya Medical College, Mangalore, IND; 3 Obstetrics and Gynecology, KIMSHEALTH, Thiruvananthapuram, IND

**Keywords:** block success, perfusion index, pulse oximetry, supraclavicular brachial plexus block, ultrasound-guided regional anesthesia

## Abstract

Background

Early and objective assessment of peripheral nerve block success remains a clinical challenge despite the widespread use of ultrasound guidance. The perfusion index (PI), derived from pulse oximetry, reflects changes in peripheral blood flow following sympathetic blockade and may serve as an early indicator of block success. This study aimed to evaluate the utility of pulse oximeter-derived PI in predicting the success of ultrasound-guided supraclavicular brachial plexus block.

Methods

This prospective observational study included 121 adult patients undergoing elective upper limb surgery under ultrasound-guided supraclavicular brachial plexus block. PI was recorded in both blocked and unblocked arms at baseline and at five-minute intervals for 30 minutes following block administration. Sensory and motor blockade were assessed using pinprick testing and the modified Bromage scale, respectively. Block success was defined by complete sensory blockade of C5-T1 dermatomes without the need for conversion to general anesthesia. Changes in PI, PI ratios, and their correlation with block success were statistically analyzed.

Results

PI in the blocked arm increased significantly from a baseline value of 2.1±0.7 to 11.0±2.3 at 30 minutes, while PI in the unblocked arm remained unchanged (p<0.0001). The PI ratio in the blocked arm rose progressively to 5.8±2.0 at 30 minutes. Successful sensory and motor blocks were achieved at 16.1±4.8 minutes and 17.1±6.4 minutes, respectively. PI demonstrated a significant positive correlation with both successful sensory block (r=0.62) and motor block (r=0.716) (p<0.0001). Overall block success was achieved in 95.9% of patients (n=116).

Conclusion

Pulse oximeter-derived PI is a simple, non-invasive, and reliable early predictor of successful ultrasound-guided supraclavicular brachial plexus block. Its routine use as an adjunct to clinical assessment may enhance the early confirmation of block success, improve perioperative efficiency, and reduce unnecessary conversion to general anesthesia.

## Introduction

Regional anesthesia has gained increasing preference for upper limb surgical procedures because it provides reliable intraoperative anesthesia and superior postoperative pain control, facilitates early mobilization, and reduces perioperative opioid requirements when compared with general anesthesia [1]. Among the various regional techniques, the supraclavicular brachial plexus block is commonly employed due to its ability to produce rapid, dense, and consistent anesthesia of the upper extremity by targeting the brachial plexus at the level of the trunks and divisions, earning it the description of the "spinal anesthesia of the upper limb" [2].

The introduction of ultrasound guidance has markedly enhanced the efficacy and safety of supraclavicular blocks by enabling the real-time visualization of neural structures, surrounding vasculature, pleura, and the spread of local anesthetic. Ultrasound-guided supraclavicular brachial plexus block has been associated with high success rates, reported to be between 90% and 98%, along with a significant reduction in complications such as pneumothorax and inadvertent vascular puncture [3,4]. Nevertheless, despite improved block performance, the early and objective determination of block success continues to pose a clinical challenge, especially in patients who are anxious, sedated, or unable to provide reliable sensory feedback [4].

Conventionally, the success of brachial plexus block is assessed using the clinical evaluation of sensory and motor blockade. Sensory block is typically evaluated using tests such as pinprick or cold sensation in the relevant dermatomal distribution, while motor blockade is assessed through the observation of muscle weakness or loss of movement in the corresponding nerve territories. In addition, patient-reported pain during surgical stimulation is often used as a practical indicator of block adequacy [5]. Moreover, they may fail to accurately reflect the onset of sympathetic blockade, which typically precedes sensory and motor block. Delayed recognition of block failure may lead to inadequate anesthesia, patient discomfort, intraoperative pain, or avoidable conversion to general anesthesia [5].

The perfusion index (PI) is a numerical parameter obtained from the pulse oximeter waveform that represents the ratio of pulsatile arterial blood flow to non-pulsatile blood flow in peripheral tissues. As PI is influenced by peripheral vascular tone, it is sensitive to alterations in sympathetic nervous system activity [6]. Regional nerve blocks produce sympathetic blockade, resulting in vasodilatation, increased peripheral blood flow, and a corresponding rise in PI in the blocked limb [6]. These hemodynamic changes typically occur within the first few minutes after successful block placement and may precede the onset of sensory and motor blockade [6].

Previous studies have reported a significant increase in PI following successful peripheral nerve blocks, often occurring within minutes after local anesthetic injection and preceding the development of clinically detectable sensory or motor blockade [7,8]. A relative increase in PI ranging from 50% to 100% above baseline has been suggested as a potential indicator of block success across different upper limb regional anesthesia techniques [8]. Compared with other objective assessment tools such as skin temperature measurement or laser Doppler flowmetry, PI monitoring offers the advantages of being non-invasive, continuous, cost-effective, and readily available using standard pulse oximetry equipment.

However, data specifically evaluating the role of PI as an early predictor of success in ultrasound-guided supraclavicular brachial plexus block remain limited, particularly in settings where access to advanced monitoring modalities is restricted. Establishing PI as a reliable and easily applicable indicator of block success may enable earlier clinical decision-making, minimize procedural delays, and improve perioperative efficiency and patient satisfaction.

Accordingly, the present study aimed primarily to evaluate the utility of pulse oximeter-derived PI as a non-invasive physiological indicator associated with the success of ultrasound-guided supraclavicular brachial plexus block. The secondary objectives were to examine the temporal relationship between PI changes and conventional clinical indicators of block onset, including sensory and motor blockade, and to analyze PI ratios and percentage changes from baseline during the early post-block period.

## Materials and methods

Study design and setting

This prospective observational study was conducted in the Department of Anesthesiology of Kasturba Hospital, Manipal, a tertiary care teaching hospital in Manipal, India, over a period of 24 months, from December 2019 to December 2021.

Study population and sample size

Adult patients aged 18-65 years with American Society of Anesthesiologists (ASA) physical status I or II, scheduled for elective upper limb surgeries distal to the shoulder under ultrasound-guided supraclavicular brachial plexus block, were included [9]. All participants were cooperative and capable of understanding the study protocol and assessment procedures.

**Figure 1:**
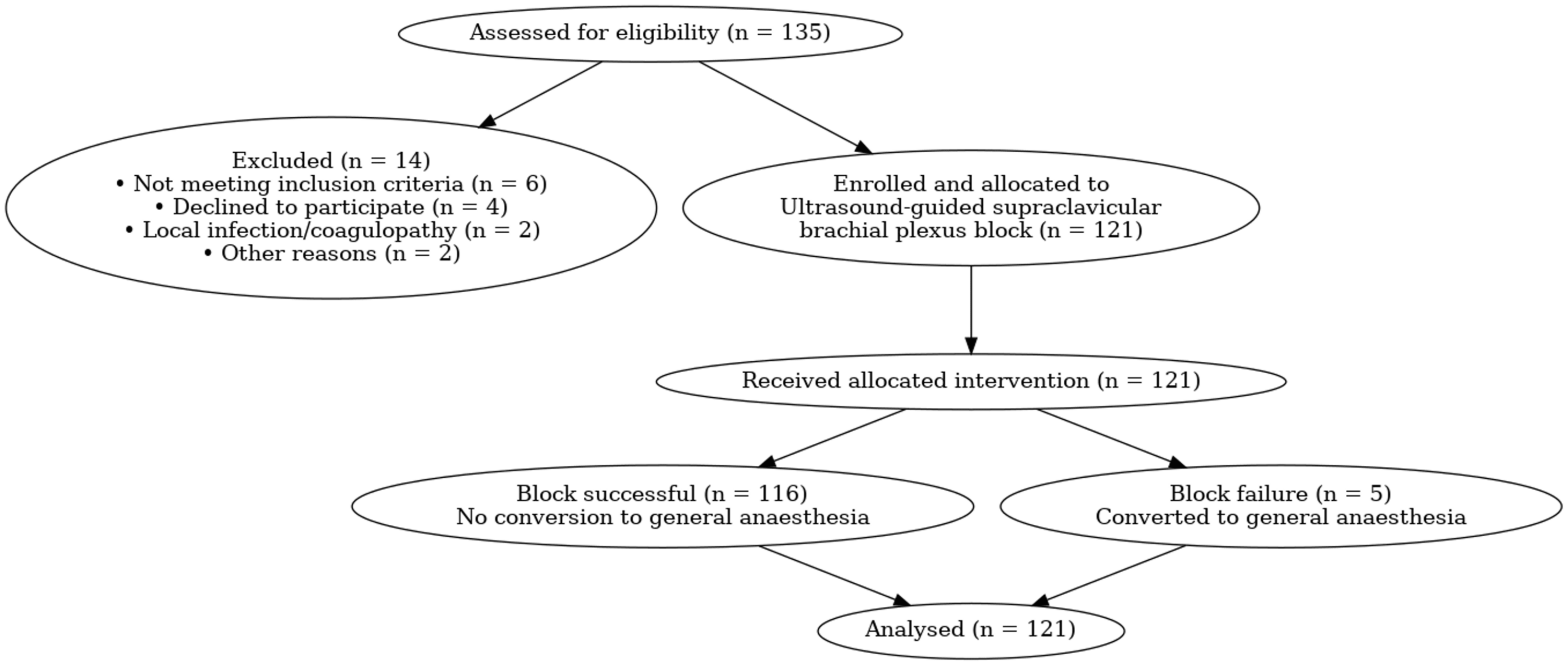
CONSORT flow diagram showing patient recruitment, allocation, follow-up, and analysis Patients were assessed for eligibility, excluded based on predefined criteria, and enrolled to receive ultrasound-guided supraclavicular brachial plexus block. Final analysis included all enrolled patients. CONSORT: Consolidated Standards of Reporting Trials

Patients with coagulopathy, infection at the injection site, pre-existing peripheral neuropathy, significant cardiovascular disease affecting peripheral perfusion, Raynaud's phenomenon, diabetes mellitus with autonomic neuropathy, allergy to local anesthetic agents, pregnancy, or refusal to participate were excluded. Patients who required conversion to general anesthesia for reasons unrelated to block performance were also excluded from the analysis (Figure 1).

Sample size was calculated based on a sensitivity-based diagnostic test formula for estimating the performance of PI in predicting successful supraclavicular brachial plexus block. Using the sensitivity reported in previous studies, an expected sensitivity of approximately 0.92 was considered. With a confidence level of 95% (Z=1.96) and an allowable error (precision) of 5%, the minimum required sample size was estimated using the formula n=Z^2^×Se×(1−Se)/d^2^ [10]. This calculation yielded a minimum sample size of approximately 113 participants. To compensate for potential exclusions, incomplete data, or block failures, an additional 10% was added, resulting in a final estimated sample size of approximately 121 patients. Therefore, at least 121 eligible patients were included in the study to ensure adequate statistical power and reliability of the results.

Preoperative preparation and procedure

Patients were evaluated on the day prior to surgery to confirm eligibility for the study. Standard preoperative fasting guidelines based on the ASA recommendations were followed, and ongoing medications were continued or withheld according to institutional protocols. On the day of surgery, patients were shifted to the preoperative holding area, and nil per os (NPO) status was reconfirmed. Standard monitoring included non-invasive blood pressure measurement, five-lead electrocardiography, and pulse oximetry using a Masimo SET® pulse oximeter (Masimo Corp., Irvine, California, United States). Intravenous access was established in the non-operative upper limb. Baseline PI values were recorded in both the operative and contralateral hands using pulse oximeter probes placed on the index fingers after stable readings were obtained.

Under strict aseptic precautions, an ultrasound-guided supraclavicular brachial plexus block was performed by an experienced anesthesiologist using a high-resolution ultrasound system (Sonosite M-Turbo®, Fujifilm Sonosite Inc., Bothell, Washington, United States) equipped with a high-frequency linear transducer. Patients were positioned in a semi-recumbent position with the head turned to the contralateral side, which improves patient comfort and facilitates ultrasound visualization of the supraclavicular brachial plexus. The brachial plexus, subclavian artery, first rib, and pleura were identified prior to needle insertion.

Using an in-plane approach, a 22-gauge insulated nerve block needle (Stimuplex®, B. Braun Melsungen AG, Germany) was advanced under real-time ultrasound visualization toward the brachial plexus. A total volume of 30 mL of local anesthetic solution, consisting of 15 mL of 0.5% bupivacaine and 15 mL of 2% lignocaine with adrenaline, was prepared in a single sterile syringe and administered incrementally after careful negative aspiration to avoid intravascular injection and to ensure adequate circumferential spread of the anesthetic around the brachial plexus. Lignocaine was administered with adrenaline in a dilution of 1:200,000 to prolong the duration of action and reduce systemic absorption. At this concentration, the vasoconstrictive effect is minimal and unlikely to significantly influence PI measurements. A combination of lignocaine and bupivacaine was used to achieve both rapid onset and prolonged duration of anesthesia. Lignocaine provides a faster onset of action due to its lower pKa and rapid nerve penetration, whereas bupivacaine offers a longer duration of sensory blockade owing to its higher protein binding and lipid solubility. The combination, therefore, allows the early establishment of block while maintaining prolonged postoperative analgesia.

Data collection

PI was continuously monitored using a Masimo SET® pulse oximeter with probes applied to the index fingers of both the blocked and contralateral limbs. Two pulse oximeter probes connected to the same monitoring system were used to obtain simultaneous readings from both hands. Baseline PI values were recorded after achieving stable readings under controlled conditions, ensuring adequate probe placement, absence of excessive patient movement, and adequate peripheral perfusion to minimize factors that could interfere with pulse oximeter reliability.

PI values were recorded at baseline and subsequently at five-minute intervals for 30 minutes following the administration of the local anesthetic. The operating room temperature was maintained between 22°C and 24°C to reduce temperature-related variations in peripheral perfusion. The PI ratio was calculated by dividing the PI measured at each time point by the baseline PI using the formula %ΔPI_t_=(PI_t_−PI_0_)/PI_0_×100.

Sensory and motor blockade were assessed at five-minute intervals for 30 minutes after block administration. Sensory blockade was evaluated using a pinprick test in the C5-T1 dermatomal distribution and graded as follows: 0: complete loss of touch sensation; 1: loss of sharp sensation and pain; 2: reduced sensitivity compared with the contralateral limb; and 3: normal sensation. Motor blockade was assessed using the modified Bromage scale for the upper limb, with grades ranging from 0 (no motor block) to 3 (complete motor block) [8]. A block was considered successful when complete sensory blockade of all C5-T1 dermatomes was achieved within 30 minutes of block administration.

The supraclavicular brachial plexus block was performed in the designated regional anesthesia block area prior to transfer to the operating room. After 30 minutes of observation and block assessment, patients were transferred to the operating room. The operating surgeon assessed the surgical site for pain using gentle pinching with surgical forceps before incision. If pain or discomfort was reported at this stage, the block was considered unsuccessful, and general anesthesia was administered.

During surgery, intravenous midazolam (1 mg) was administered for anxiolysis when required. If patients experienced pain suggestive of incomplete or patchy block, rescue analgesia with intravenous fentanyl (1 µg/kg) was administered. Persistent pain despite rescue analgesia resulted in conversion to general anesthesia at the discretion of the attending anesthesiologist. Two independent observers participated in the study to minimize bias. An anesthesia postgraduate resident conducted the pre-anesthetic evaluation, recorded PI values, and calculated PI ratios. An experienced anesthesia consultant performed the ultrasound-guided supraclavicular brachial plexus block.

Statistical analysis

Data were entered into Microsoft Excel (Microsoft Corporation, Redmond, Washington, United States) and analyzed using IBM SPSS Statistics for Windows, Version 22.0 (IBM Corp., Armonk, New York, United States). Continuous variables were expressed as mean±standard deviation or median with interquartile range, as appropriate. Categorical variables were summarized as frequencies and percentages. Changes in PI over time were analyzed using repeated-measures analysis of variance with Bonferroni post hoc correction. Pearson's correlation coefficient was used to assess the relationship between PI changes and block success. A p-value of <0.05 was considered statistically significant.

Ethical consideration

Approval was obtained from the Institutional Ethics Committee of Kasturba Medical College and Kasturba Hospital, Manipal (approval number: 920/2017; date: September 25, 2019). The study was registered in the Clinical Trials Registry-India (CTRI) under the registration number CTRI/2019/01/017187 dated January 20, 2019. Moreover, it was conducted in accordance with the ethical principles outlined in the Declaration of Helsinki. Written informed consent was obtained from all participants prior to enrolment.

## Results

A total of 121 patients were included in the study. The mean age of participants was 36.3±13.1 years, with the majority belonging to the 21-30-year age group (42, 34.7%), followed by the 51-60-year age group (27, 22.3%). Male patients constituted 69.4% (n=84) of the study population. The mean body weight and height were 67.5±9.3 kg and 166.1±10.9 cm, respectively, with a mean body mass index of 24.4±2.2 kg/m². Baseline vital parameters were within normal physiological limits, including the mean heart rate (76.4±9.8 beats/min), systolic blood pressure (125.7±17.7 mmHg), diastolic blood pressure (70.9±11.4 mmHg), and oxygen saturation (98.7±1.2%) (Table 1).

**Table 1:** Baseline demographic and clinical characteristics of the study population BMI: body mass index; SpO₂: peripheral oxygen saturation

Variables	Frequency (%)/mean±SD
Age group (years)	
≤20	12 (9.9%)
21-30	42 (34.7%)
31-40	22 (18.2%)
41-50	18 (14.9%)
51-60	27 (22.3%)
Age (years)	36.3±13.1
Gender	
Female	37 (30.6%)
Male	84 (69.4%)
Weight (kg)	67.5±9.3
Height (cm)	166.1±10.9
Body mass index (kg/m²)	24.4±2.2
Heart rate (beats/min)	76.4±9.8
Systolic blood pressure (mmHg)	125.7±17.7
Diastolic blood pressure (mmHg)	70.9±11.4
SpO₂ (%)	98.7±1.2

Baseline PI values were comparable between the blocked arm (2.1±0.7) and the unblocked arm (2.0±0.6). Baseline measurements were obtained prior to block administration and therefore included all enrolled participants, including those who subsequently required conversion to general anesthesia. Following the administration of the supraclavicular brachial plexus block, the PI in the blocked arm showed a progressive and significant increase over time, reaching 11.0±2.3 at 30 minutes. In contrast, the PI in the unblocked arm remained relatively unchanged throughout the observation period. The difference in PI trends between the blocked and unblocked arms was statistically significant (p<0.0001) (Table 2).

**Table 2:** Changes in perfusion index over time between the blocked and unblocked arms T0-T30: time in minutes after block administration

Perfusion index	Blocked arm	Unblocked arm
	Mean±SD	
T0	2.1±0.7	2.0±0.6
T5	3.6±1.0	2.0±0.6
T10	5.5±1.5	2.0±0.6
T15	7.2±1.8	2.1±0.6
T20	8.6±1.9	2.1±0.6
T25	9.9±2.0	2.1±0.6
T30	11.0±2.3	2.1±0.6
P-value	<0.0001	<0.0001

The PI ratio (PI at time T divided by baseline PI) increased markedly in the blocked arm, with mean values rising from 1.8±0.5 at five minutes to 5.8±2.0 at 30 minutes. In contrast, the unblocked arm demonstrated minimal change in PI ratio across all time points, remaining close to baseline. The difference in PI ratio between the blocked and unblocked arms was statistically significant (p<0.0001) (Table 3).

**Table 3:** Perfusion index ratio (T/T0) between the blocked and unblocked arms T0-T30: time in minutes after block administration

Perfusion index ratio	Blocked arm	Unblocked arm
	Mean±SD	
T5 (T5/T0)	1.8±0.5	1.1±0.7
T10 (T10/T0)	2.8±1.0	1.0±0.1
T20 (T20/T0)	4.5±1.6	1.0±0.1
T30 (T30/T0)	5.8±2.0	1.0±0.1
P-value	<0.0001	<0.0001

Motor blockade progressed steadily over time, with higher grades becoming increasingly prevalent. At 15 minutes, 60.3% of patients (n=73) had achieved complete motor block (Level 3), which increased to 94.2% (n=114) by 30 minutes. Correspondingly, the mean motor block score increased from 0.9±0.5 at five minutes to 2.9±0.4 at 30 minutes. Sensory blockade showed an earlier onset compared to motor blockade. At five minutes, the majority of patients were at sensory Level 2 (93, 76.9%), and by 20 minutes, 87.6% (n=106) had achieved complete sensory loss (Level 0). At 30 minutes, 96.7% of patients (n=117) demonstrated complete sensory blockade. The mean sensory block score progressively decreased from 2.0±0.5 at five minutes to 0.1±0.4 at 30 minutes (Table 4).

**Table 4:** Temporal distribution of motor and sensory blockade in the blocked arm Motor block was graded using the modified Bromage scale (0-3). Sensory block was graded from 0 (complete loss) to 3 (normal sensation).

Blocked arm	Level 0	Level 1	Level 2	Level 3	Mean±SD
	Frequency (%)				
Motor block					
T5	23 (19%)	85 (70.2%)	13 (10.7%)	0 (0%)	0.9±0.5
T10	5 (4.1%)	27 (22.3%)	57 (47.1%)	32 (26.4%)	2.0±0.8
T15	1 (0.8%)	12 (9.9%)	35 (28.9%)	73 (60.3%)	2.5±0.7
T20	0 (0%)	5 (4.1%)	19 (15.7%)	97 (80.2%)	2.8±0.5
T25	0 (0%)	5 (4.1%)	6 (5%)	110 (90.9%)	2.9±0.4
T30	0 (0%)	5 (4.1%)	2 (1.7%)	114 (94.2%)	2.9±0.4
Sensory block					
T5	0 (0%)	14 (11.6%)	93 (76.9%)	14 (11.6%)	2.0±0.5
T10	28 (23.1%)	66 (54.5%)	27 (22.3%)	0 (0%)	1.0±0.7
T15	77 (63.6%)	35 (28.9%)	9 (7.4%)	0 (0%)	0.4±0.6
T20	106 (87.6%)	11 (9.1%)	4 (3.3%)	0 (0%)	0.2±0.4
T25	114 (94.2%)	3 (2.5%)	4 (3.3%)	0 (0%)	0.1±0.4
T30	117 (96.7%)	0 (0%)	4 (3.3%)	0 (0%)	0.1±0.4

A supraclavicular brachial plexus block was considered successful when complete sensory blockade of all C5-T1 dermatomes was achieved within 30 minutes of block administration and the surgical procedure was completed without the need for conversion to general anesthesia. An unsuccessful block was defined as failure to achieve complete sensory blockade within 30 minutes of block administration or the presence of pain during surgical stimulation requiring conversion to general anesthesia. A successful supraclavicular brachial plexus block was achieved in the majority of patients, with 95.9% (n=116) not requiring conversion to general anesthesia. The mean time to achieve successful sensory block was 16.1±4.8 minutes, while successful motor block was achieved at 17.1±6.4 minutes. The mean PI at the time of successful sensory and motor block was 7.4±1.8 and 7.9±3.0, respectively. Supplemental analgesia was required in 12.4% (n=15) of patients during surgery (Table 5).

**Table 5:** Block success, anesthetic requirements, and supplemental analgesia

Variable	Frequency (%)/mean±SD
General anesthesia required	
No	116 (95.9%)
Yes	5 (4.1%)
Time to successful sensory block (min)	16.1±4.8
Time to successful motor block (min)	17.1±6.4
Perfusion index at successful sensory block	7.4±1.8
Perfusion index at successful motor block	7.9±3.0
Supplemental analgesia required	
No	106 (87.6%)
Yes	15 (12.4%)

**Figure 2:**
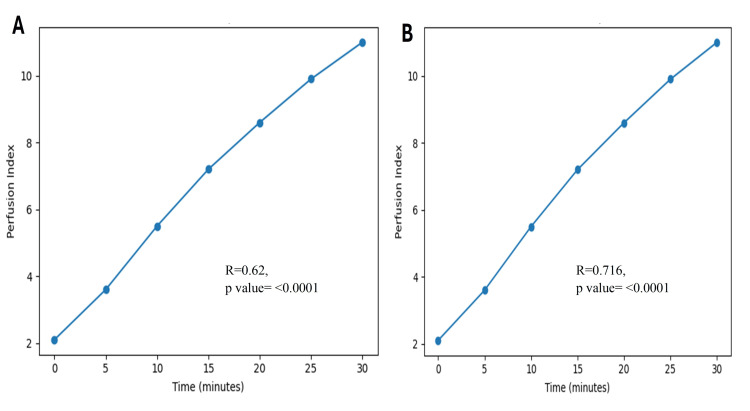
(A) Perfusion index change over time and correlation with successful sensory block. (B) Perfusion index change over time and correlation with successful motor block

PI changes demonstrated a statistically significant positive correlation with both sensory and motor block success. Pearson's correlation coefficient between PI change and successful sensory block was r=0.62, while a stronger correlation was observed with successful motor block (r=0.716). Both correlations were highly significant (p<0.0001) (Table 6 and Figure 2).

**Table 6:** Correlation between perfusion index changes and block success (n=116)

Variables	Perfusion index changes	P-value
	Pearson's correlation coefficient	
Successful sensory block	0.62	<0.0001
Successful motor block	0.716	<0.0001

## Discussion

The findings of the present study indicate that the pulse oximeter-derived PI serves as a dependable and objective early marker of successful ultrasound-guided supraclavicular brachial plexus block. The consistent and statistically significant increase in PI observed in the blocked limb, alongside the absence of comparable changes in the contralateral limb, supports the physiological premise that PI reflects sympathetic blockade-induced vasodilatation and enhanced peripheral blood flow [11,12].

The baseline demographic and hemodynamic characteristics of the study population were comparable to those reported in previous studies by Albrecht et al. and Park et al., conducted in elective upper limb surgery settings, with a predominance of younger male patients [13,14]. Stable pre-block heart rate, blood pressure, and oxygen saturation values in the present cohort suggest that the observed alterations in PI were predominantly attributable to regional sympathetic blockade rather than systemic hemodynamic variations, thereby reinforcing the internal validity of the results [15,16].

The early increase in PI observed after block administration can be attributed to sympathetic blockade produced by local anesthetic agents. By inhibiting sodium channel conduction in sympathetic nerve fibers, these agents induce vasodilatation in the blocked limb, leading to increased peripheral blood flow and enhanced pulsatile signals detected by pulse oximetry. The rapid onset of lignocaine and the longer duration of bupivacaine may together contribute to the progressive rise in PI following successful block placement. A notable observation in this study was the early onset and progressive elevation of PI in the blocked arm, evident within five minutes of block administration and increasing nearly fivefold by 30 minutes. The monitoring period was limited to the initial 30 minutes to capture early perfusion changes associated with block onset; therefore, regression of PI during the recovery phase of the block was not evaluated in this study. In contrast, PI values in the unblocked arm remained largely unchanged throughout the observation period. This limb-specific response further confirms that PI is a marker of localized sympathetic denervation rather than generalized circulatory effects [17]. Similar trends have been documented by Veena et al. and Rajeev et al., who reported that sympathetic blockade precedes clinically detectable sensory and motor blockade and is associated with early increases in peripheral perfusion measurable using pulse oximetry-based indices [18,19].

Assessment of the PI ratio (PIₜ/PI₀) further strengthened these findings by accounting for inter-individual variability in baseline PI values. In the present study, the PI ratio in the blocked limb demonstrated a steady rise, reaching 5.8±2.0 at 30 minutes, whereas values in the unblocked limb remained close to baseline. In contrast, patients with unsuccessful blocks did not show a comparable increase in PI ratio, likely reflecting inadequate sympathetic blockade and minimal change in peripheral perfusion. These observations support the concept that relative changes in PI provide greater clinical insight than absolute values alone. Comparable results have been reported in studies by Junghare et al. and Jain et al. evaluating the role of PI in infraclavicular and interscalene brachial plexus blocks [20,21]. The highly significant difference between limbs (p<0.0001) highlights the robustness of the PI ratio as a predictor of block success.

The temporal relationship between PI changes and clinical block characteristics is of particular clinical importance. In this study, sensory blockade was achieved earlier than motor blockade, with most patients attaining complete sensory block by 20 minutes, while motor block developed more gradually. This pattern is consistent with established neurophysiological principles, wherein smaller, lightly myelinated sympathetic and sensory fibers are more susceptible to local anesthetic action than larger, heavily myelinated motor fibers [22,23]. The concurrent rise in PI during this early phase further emphasizes its utility as an early indicator of block success, potentially enabling clinicians to anticipate adequate block onset before conventional sensory or motor testing becomes conclusive [24].

The significant positive correlations observed between PI changes and both sensory and motor block success further validate the predictive value of PI. The stronger correlation with motor block success (r=0.716) compared to sensory block success (r=0.62) may reflect the more extensive and sustained sympathetic and somatic blockade required for dense motor block, resulting in greater peripheral vasodilatation [25]. Similar correlation strengths have been reported by Mahajan et al. and Abdelhamid et al., who evaluated PI and skin temperature as indicators of peripheral nerve block efficacy [26,27]. However, PI offers the added advantages of continuous monitoring, non-invasiveness, and widespread availability on standard pulse oximetry platforms.

From a clinical perspective, the high overall block success rate observed in this study (95.9%) and the low requirement for conversion to general anesthesia or supplemental analgesia underscore the effectiveness of ultrasound-guided supraclavicular brachial plexus block. Importantly, the mean PI values observed at the time of successful sensory and motor block (approximately 7.4 and 7.9, respectively) provide clinically relevant reference points that may assist anesthesiologists in early decision-making [28]. Early recognition of inadequate or absent PI elevation could facilitate the prompt identification of ineffective blocks and allow timely intervention, thereby minimizing intraoperative discomfort, procedural delays, and unnecessary exposure to general anesthesia [29]. However, PI changes should be interpreted in conjunction with the clinical assessment of sensory and motor blockade.

Compared with conventional block assessment methods such as pinprick testing and motor evaluation, which are subjective, intermittent, and dependent on patient cooperation, PI offers an objective and observer-independent measure [30]. This characteristic is particularly valuable in sedated, anxious, or non-communicative patients, where traditional assessments may be unreliable. Furthermore, as PI monitoring requires no additional equipment beyond routine pulse oximetry, it represents a practical and cost-effective adjunct in both high-resource and resource-limited clinical settings [30].

Limitations

Certain limitations of this study should be acknowledged. The single-center design and relatively modest sample size may limit the generalizability of the findings. PI measurements are susceptible to external influences such as ambient temperature, probe placement, and patient movement, despite attempts to standardize monitoring conditions. Additionally, anatomical variations in the brachial plexus or surrounding vascular structures may influence the distribution of local anesthetics and may contribute to patchy blocks if not carefully visualized during ultrasound-guided block placement. Finally, while PI provides an objective indicator of sympathetic blockade, it does not directly measure sensory or motor nerve function and should be considered a complementary tool rather than a substitute for conventional clinical assessment.

## Conclusions

The present study suggests that pulse oximeter-derived PI may serve as a useful, non-invasive, and early physiological marker associated with the onset of ultrasound-guided supraclavicular brachial plexus block. PI values were obtained directly from the pulse oximeter monitor without the need for additional calculations, and measurements were recorded at baseline and at five-minute intervals following block administration. In the present study, a progressive increase in PI was observed in the blocked limb within the first five minutes and continued to rise over the 30-minute observation period, paralleling the development of sensory and motor blockade. These findings suggest that changes in PI may reflect sympathetic blockade and the associated vasodilatation occurring during block onset. Although PI changes showed a clear association with successful block development, they should be interpreted alongside clinical assessment rather than used as a standalone indicator of block success. No universal cut-off value for PI has been established for predicting successful brachial plexus block; however, several studies have suggested that a significant relative increase from baseline may indicate effective sympathetic blockade. Therefore, PI monitoring may complement conventional sensory and motor block evaluation by providing an early, objective indicator of block progression and facilitating the timely recognition of inadequate block development. Given that PI measurement is simple, non-invasive, and readily available on standard monitoring devices, it may serve as a useful adjunct to clinical assessment, particularly in sedated or non-communicative patients.
